# Evolution of external female genital mutilation: why do males harm their mates?

**DOI:** 10.1098/rsos.171195

**Published:** 2017-11-01

**Authors:** Pierick Mouginot, Gabriele Uhl, Lutz Fromhage

**Affiliations:** 1 General and Systematic Zoology, Zoological Institute and Museum, University of Greifswald, Greifswald 17489, Germany; 2 Department of Biological and Environmental Science, University of Jyväskylä, PO Box 35, Jyväskylä 40014, Finland

**Keywords:** sexual selection, sperm competition, harmful male trait, genital damage, mating costs

## Abstract

Sperm competition may select for male reproductive traits that influence female mating or oviposition rate. These traits may induce fitness costs to the female; however, they may be costly for the males as well as any decrease in female fitness also affects male fitness. Male adaptations to sperm competition manipulate females by altering not only female behaviour or physiology, but also female morphology. In orb-weaving spiders, mating may entail mutilation of external structures of the female genitalia, which prevents genital coupling with subsequent males. Here, we present a game theoretical model showing that external female genital mutilation is favoured even under relatively high costs of mutilation, and that it is favoured by a high number of mate encounters per female and last-male sperm precedence.

## 1. Introduction

Males may evolve traits that shift the remating or oviposition rate of the female from the female's optimum towards their own due to selection on competitive fertilization success [1,2]. Defensive adaptations to sperm competition include mate guarding, copulatory plugs, manipulative seminal fluids and internal genital damage. These adaptations can manipulate the female by altering her behaviour or physiology, but also her genital anatomy [3]. Examples of male-inflicted damages to female genitalia have been documented in numerous taxa, especially among arthropods [4,5]. Most of these genital damages are inflicted internally by the male intromittent organ, and it is debated how these harmful traits have evolved [6–8].

Recently, it has been described that males inflict external damage to the female genitalia in two species of orb-weaving spiders as a defensive adaptation to sperm competition [9,10]. In the course of copulation, males mutilate an external genital structure of the female that is necessary for genital coupling. The lack of this structure prevents the female from remating [9]. Although probably widespread among spiders [9], the selection regime for external female genital mutilation (EFGM) is difficult to understand, as any decrease in female fitness caused by the mutilation should also reduce the male's fitness [1]. Here, we present a game theoretical model to explore the conditions under which EFGM can evolve and be maintained.

## 2. Model and results

We consider an infinite population with a sex ratio at maturity of *r* males per female. During a mating season, each female experiences a number *n* of mate encounters with different males. In the absence of genital mutilation, females mate with every male they encounter. After that, the female lays eggs and dies. For simplicity, *n* does not vary between females. Because every mate encounter involves a male and a female, the total number of encounters must be the same for both sexes, implying that each male encounters on average *n/r* females. We consider two mating strategies for males: ‘harmless’ males do not perform mutilation of the female genitalia during copulation, nor do they prevent the females from remating. ‘Mutilator’ males damage every female they mate with. This reduces female fitness by proportion *α* (the cost of mutilation) and prevents females from remating with subsequent males. Following a game theoretic approach [11], we seek conditions where each strategy can invade (i.e. is favoured by selection when rare) and is an evolutionarily stable strategy (ESS; a strategy which, when common, cannot be invaded by the alternative strategy). For this purpose, we compare the fitness of a rare ‘mutant’ strategy to the fitness of the ‘resident’ strategy adopted by the majority of the population. We consider the case where *mutilator* is the mutant strategy and *harmless* is the resident strategy, and vice versa. In doing so, we follow the standard assumption that the mutant strategy is so rare that its effect on the resident strategy's fitness is negligible. See table 1 for a summary of the variables.

**Table 1.** Summary of the model variables.

variable	meaning	constraint
*r*	sex ratio at maturity	
*n*	number of mate encounters per female (with different males)	
*α*	cost of mutilation as a proportion of female fitness	0 ≤ *α *≤ 1
*i*	male's position in the female mating sequence	
*L*	‘loading factor’; characterizes the sperm precedence strength. Sperm precedence is absent if *L *= 1	*L* ≥ 1
*p*	paternity share	0 ≤ *p* ≤ 1

### 2.1. Each female encounters only a single male

We begin by establishing a general result that holds regardless of patterns of sperm precedence. Consider the case where each female encounters only a single male (*n *= 1) and consequently each encounter leads to 100% paternity. Defining the fitness of an unmutilated female as 1, and expressing male fitness in relation to this, the fitness *W*_H_ of a *harmless* male is then equivalent to his number of mate encounters, *n/r*

2.1 $$W_{H}=\frac{n}{r}\frac{1}{n}=\frac{1}{r}.$$

Compared to this, the fitness *W*_M_ of a *mutilator* is reduced by the cost of mutilation (*α*)

2.2 $$W_{M}=\frac{n}{r}\frac{1}{n}(1-\alpha )=\frac{1}{r}(1-\alpha ).$$

It is a special property of the *n *= 1 case that the fitnesses of both strategies do not depend on which strategy is currently rare or common in the population. Thus, substituting equations (2.1) and (2.2) into *W*_H_ > *W*_M_, we obtain

2.3 α>0,

as the condition where *harmless* males have higher fitness than *mutilators* at any frequency. This means that in the *n *= 1 case, mutilation is selected against whenever it imposes any cost on females.

### 2.2. Each female encounters more than one male

#### 2.2.1. Mutilator invasion

We consider the case where each female encounters more than one male (*n* > 1) and the resident strategy is *harmless*. As the female will mate with all *n* males she encounters, the paternity over a female's offspring will be shared among her mates. Here, we consider the possibility that either the first or the last male may enjoy an advantage in sperm competition. A *harmless* male has a probability 1*/n* of obtaining the position in a female's mating sequence that grants him sperm precedence, providing him with the paternity share

2.4 $$p[i]=\frac{L}{\left(L+i-1\right)},$$

when mating with a female that mates *i* times in total. This formulation is called a ‘loaded raffle’ [12], in which the priority male's sperm has *L* times higher competitive weight than his competitors' sperm. Parameter *L*, called a ‘loading factor’, characterizes the sperm precedence strength and satisfies *L* ≥ 1. The absence of sperm precedence (also called a ‘fair raffle’ process) is included in the formulation as the special case, where *L *= 1.

The paternity of a *harmless* male is then *p*[*n*], because the female will mate with all *n* males she encounters. In every encounter, he also has a probability (*n *− 1)/*n* of not obtaining sperm precedence, instead being one of the *n *− 1 males that share the remaining paternity, 1 − *p*[*n*]. The fitness *W*_H_ of a *harmless* resident male is therefore:

2.5 $$W_{H}=\frac{n}{r}\left(\frac{1}{n}p[n]+\frac{n-1}{n}\left(\frac{1-p[n]}{n-1}\right)\right)=\frac{1}{r}.$$

While this is independent of *p*[*n*], and hence of sperm precedence, sperm precedence becomes important when calculating the fitness of *mutilator* mutants in this population.

##### 2.2.1.1. First-male precedence

In each of his *n*/*r* mate encounters, a *mutilator* mutant has a probability 1/*n* of being the *i*th male to encounter (and mate with) a given female. In each of his matings, a mutilator prevents the female from remating with subsequent males. Therefore, as the first male, he gets 100% of the paternity. And as the *i*th male (where *i *> 1), he limits the female to *i* matings in total, and is hence one of *i *− 1 males that share the paternity (1 − *p*[*i*]) left over by the first male. The fitness of a *mutilator* mutant is therefore:

2.6 $$W_{M}=\frac{n}{r}\frac{1}{n}\left(1+\sum_{i=2}^{n}\frac{1-p[i]}{i-1}\right)(1-\alpha ).$$

Substituting the fitness (equations (2.5) and (2.6)) and paternity share (equation (2.4)) equations into *W*_M_ > *W*_H_, we obtain

**Figure 1.**
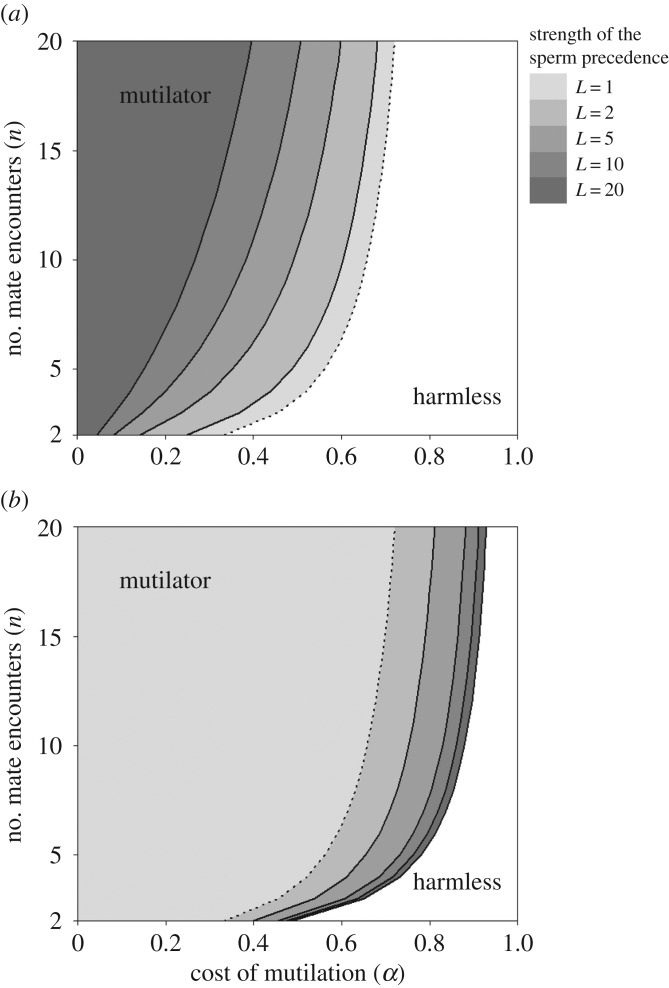
ESS regions under first-male (*a*) and last-male (*b*) sperm precedence in parameter space of the cost of mutilation (*α*) and the number of mate encounters per female (*n*). The curves represent the limits of the ESS regions for different sperm precedence strengths: *L *= 1, 2, 5, 10, 20, where *L *= 1 (dashed line) represents the situation with no sperm precedence. To the right of each curve, the *harmless* strategy is an ESS. To the left of each curve, the *mutilator* strategy can invade, and is then also an ESS. (*a*) The ESS region reduces towards darker shaded areas when the strength of the sperm precedence increases. (*b*) The ESS region extends towards darker shaded areas when the strength of the sperm precedence increases.

2.7 $$(1-\alpha )\left(1+\sum_{i=2}^{n}\frac{1}{L+i-1}\right)>1,$$

as the condition for which the *mutilator* strategy can invade. This corresponds to the area of parameter space illustrated in figure 1*a*.

##### 2.2.1.2. Last-male precedence

In each of his matings, a *mutilator* mutant will be the last of the female's mates, thus securing the position that grants him the last-male sperm competition advantage. In each of his *n*/*r* mate encounters, a *mutilator* mutant has a probability 1/*n* of being the *i*th male to encounter (and mate with) a given female. In any case, he will be the last of the female's mates, thus, securing the position that grants him the last-male sperm competition advantage, he will receive paternity *p*[*i*]. The fitness of a *mutilator* mutant is therefore:

2.8 $$W_{M}=\frac{n}{r}\frac{1}{n}\sum_{i=1}^{n}p[i](1-\alpha ).$$

Substituting the fitness (equations (2.5) and (2.8)) and paternity share (equation (2.4)) equations into *W*_M_ > *W*_H,_ we obtain

2.9 $$(1-\alpha )\sum_{i=1}^{n}\frac{L}{L+i-1}>1,$$

as the condition where the *mutilator* strategy can invade. This corresponds to the area of parameter space illustrated in figure 1*b*.

### 2.3. Mutilator stability

We now consider the case where each female encounters more than one male (*n *> 1) and *mutilator* is the resident strategy. In this situation, females are mutilated at their first mating and *mutilators* obtain full paternity. Therefore, a male only gets any paternity when he encounters a virgin female, as happens in 1/*n* of his encounters. If a *harmless* male enters the population of *mutilator* males and mates with a virgin female, the female can still suffer the cost of mutilation from a subsequent mating with a *mutilator*. The fitness of a *harmless* mutant is thus

2.10 $$W_{H}=\frac{n}{r}\frac{1}{n}p(1-\alpha )=\frac{p(1-\alpha )}{r},$$

where *p* is the *harmless* mutant's paternity share. The cost of mutilation is present in the fitness expression of *W*_H_ (equation (2.10)) because of the subsequent mating by a *mutilator*. By contrast, the fitness of a resident *mutilator* is

2.11 $$W_{M}=\frac{n}{r}\frac{1}{n}(1-\alpha )=\frac{1-\alpha }{r},$$

because *mutilators* obtain full paternity when mating with virgin females. Thus, substituting the fitness equations (equations (2.10) and (2.11)) into *W*_M_ > *W*_H_, we obtain

2.12 p<1,

as the condition where *mutilator* is an ESS. Therefore, *mutilator* is an ESS as long as the *harmless* mutant does not obtain full paternity (i.e. *p* < 1). This is true whenever a subsequent mating reduces the first male's paternity below 100%, regardless of *n* and the exact pattern of sperm precedence. This includes, but goes far beyond, all the conditions where the *mutilator* strategy can invade (see above). In other words, the conditions under which *mutilator* is stable are much broader than the conditions under which it can invade.

## 3. Discussion

Our model predicts that the evolution of EFGM can evolve even under relatively high costs of mutilation (*α*), and that it is favoured by a high number of mate encounters per female (*n*) and ancestral last-male sperm precedence (figure 1*a*,*b*).

The cost of mutilation is an assumption of the model. It characterizes any cost that is possibly incurred after a physical damage as wound healing, increased immune response or infection risk [13–16]. Although there is currently no evidence for EFGM reducing female fitness, this absence of costs may be the result of selection on females for reducing such costs [1,2,17]. However, when it first evolved, EFGM was probably costly because females had not yet evolved any counteradaptation. The role of mutilation costs in this context is straightforward: for any given paternity share that a *mutilator* might attain through his matings, his resultant number of offspring is proportional to the number of offspring produced by his mates. Thus, other things being equal, increasing the mutilation cost (α) reduces the extent to which *mutilators* can benefit from their behaviour, up to the point of making it impossible for them to invade. Even though the prediction is that a strategy is more likely to evolve for low costs, EFGM is still beneficial up to relatively high costs (figure 1*a*,*b*). The limiting aspect of mutilation costs concurs with the theory predicting that harmful males can be favoured provided that the benefits from harming their mates outweigh the costs of reducing their mate's offspring production [1]. Once *mutilators* are common, however, and all females are mutilated sooner or later (because *n > *1), then mutilation costs no longer reduce the fitness of *mutilators* compared with *harmless* males. This explains why the *mutilator* strategy is stable under much broader conditions that those that allow its invasion.

Taken together, the result that EFGM is stable once it evolves, and the conjecture that mutilation costs are reduced over evolutionary time, leads to another prediction: regardless of current mutilation costs, we expect EFGM to occur more frequently in species in which EFGM invasion would have been possible even under high ancestral costs of mutilation.

Our model shows the evolution of EFGM to be facilitated by a high number of mate encounters per female (*n*) (figure 1*a*,*b*). This is because the mutilation increases a *mutilator*'s paternity only in situations where the female will encounter at least one subsequent male in the future, who then cannot mate as a result of the mutilation. By contrast, if a *mutilator* is anyway the last male to encounter a given female, then the mutilation merely causes unnecessary damage. This maladaptive outcome occurs in a proportion 1/*n* of mate encounters, and hence becomes more likely if *n* is small. It is worth noting that it does not matter for this argument if males are monogamous or polygynous. For example, *n* = 2 could apply if the sex ratio is even and males mate twice, or if the sex ratio is male-biased and males mate only once.

Harmful adaptation to sperm competition should be more common in populations with last-male sperm precedence because the risk of a decreased sperm competition success due to the female remating amplifies the advantage of securing the female [18]. Indeed, EFGM invades more easily if the sperm precedence pattern is last-male precedence (figure 1*b*). This occurs because *mutilators* always secure the position of ‘last male’, which is particularly advantageous under last-male sperm precedence. By contrast, first-male precedence hinders *mutilator* invasion (figure 1*a*) because preventing female remating is less advantageous if subsequent males obtain little paternity. We predict that EFGM occurrence across spider groups is associated with current or ancestral last-male sperm precedence.

While our predictions do not explicitly depend on the sex ratio at maturation (*r*), it would be misleading to conclude from this that *r* is irrelevant in the context of EFGM. In fact, our model merely predicts that *r* has no additional (independent) effect for a given number of mate encounters per female (*n*). This does not rule out the possibility that the sex ratio has an effect *via* the number of mate encounters. Indeed, other things being equal, increasing the number of males per female should also increase the mate encounters per female. However, mate encounters may also depend on many other factors, including population density, habitat structure, movement ability, male mortality during mate search, male sperm limitation, as well as the timing of maturation. While we did not model these factors explicitly, they are implicitly accounted for insofar as they affect the number of mate encounters per female (*n*).

Once EFGM has evolved, it is likely to have further evolutionary consequences that are not captured by our model. For example, as EFGM makes it beneficial to mate with virgin females, it may select for protandry (i.e. males maturing before females). Interestingly, because protandry is generally expected in species with first-male precedence [19], but (ancestral) first-male precedence hinders the evolution of EFGM (figure 1*a*), this suggests a particular sequence of evolutionary events: if protandry is found in species with EFGM, then our model suggests that EFGM evolved before protandry. However, it is also possible that protandry increases the sex ratio at maturation, which then increases the mate encounters per female, thereby facilitating the evolution of EFGM (figure 1*a*,*b*). Either way, we would empirically expect to find a positive association between EFGM and protandry.

While our present model has focused on the evolution of male behaviour, EFGM should also select for evolutionary responses in females. There are two main ways in which females could adapt to EFGM: resistance or tolerance [1,2,17,20,21]. By evolving resistance traits such as mutilation avoidance behaviour or more sclerotized genitalia, females may avoid EFGM along with the associated costs. Alternatively, females may evolve tolerance traits (or ‘palliative adaptations’ [17]), such as modified genitalia, that reduce (and eventually eliminate) the costs of mutilation, without preventing EFGM as such. While it seems difficult to predict which of these pathways is more likely to occur, the pathway taken should affect the probability that EFGM can in fact be observed: if females evolve resistance, this will tend to eliminate the evidence that EFGM ever existed. By contrast, if females evolve to minimize the associated costs, EFGM may readily be observed in the long run. Another evolutionary response that is worthy of investigation is how EFGM affects the evolution of female mate choice. However, as our focus here has been on male decisions, it is beyond the scope of our study.

## Data accessibility

As this is a theoretical work, this does not apply to our study.
